# Distinctive Electric Properties of Group 14 Oxides: SiO_2_, SiO, and SnO_2_

**DOI:** 10.3390/ijms242115985

**Published:** 2023-11-05

**Authors:** Antonio Nuno Guerreiro, Ilidio B. Costa, Antonio B. Vale, Maria Helena Braga

**Affiliations:** 1 Engineering Physics Department, Engineering Faculty, University of Porto, 4200-465 Porto, Portugal; nguerreiro@fe.up.pt; 2 MatER—Materials for Energy Research Laboratory, Engineering Faculty, University of Porto, 4200-465 Porto, Portugal; up202006411@edu.fe.up.pt (I.B.C.); up201709900@edu.fe.up.pt (A.B.V.); 3 Metallurgical and Materials Engineering Department, Engineering Faculty, University of Porto, 4200-465 Porto, Portugal; 4 LAETA—INEGI, Institute of Science and Innovation in Mechanical and Industrial Engineering, 4200-465 Porto, Portugal

**Keywords:** oxides group 14, silica, silicon monoxide, tin dioxide, scanning kelvin probe, work functions, ab initio simulations, electronic band structure, charge carrier mobilities

## Abstract

The oxides of group 14 have been widely used in numerous applications in glass, ceramics, optics, pharmaceuticals, and food industries and semiconductors, photovoltaics, thermoelectrics, sensors, and energy storage, namely, batteries. Herein, we simulate and experimentally determine by scanning kelvin probe (SKP) the *work functions* of three oxides, SiO_2_, SiO, and SnO_2_, which were found to be very similar. Electrical properties such as electronic band structure, electron localization function, and carrier mobility were also simulated for the three crystalline oxides, amorphous SiO, and surfaces. The most exciting results were obtained for SiO and seem to show Poole–Frankel emissions or trap-assisted tunneling and propagation of surface plasmon polariton (SPP) with nucleation of solitons on the surface of the Aluminum. These phenomena and proposed models may also describe other oxide-metal heterojunctions and plasmonic and metamaterials devices. The SiO_2_ was demonstrated to be a stable insulator interacting less with the metals composing the cell than SnO_2_ and much less than SiO, configuring a typical Cu/SiO_2_/Al cell potential well. Its surface charge carrier mobility is small, as expected for an insulator. The highest charge carrier mobility at the lowest conduction band energy is the SnO_2_’s and the most symmetrical the SiO’s with a similar number of electron holes at the conduction and valence bands, respectively. The SnO_2_ shows it may perform as an n-type semiconductor.

## 1. Introduction

**Figure 1:**
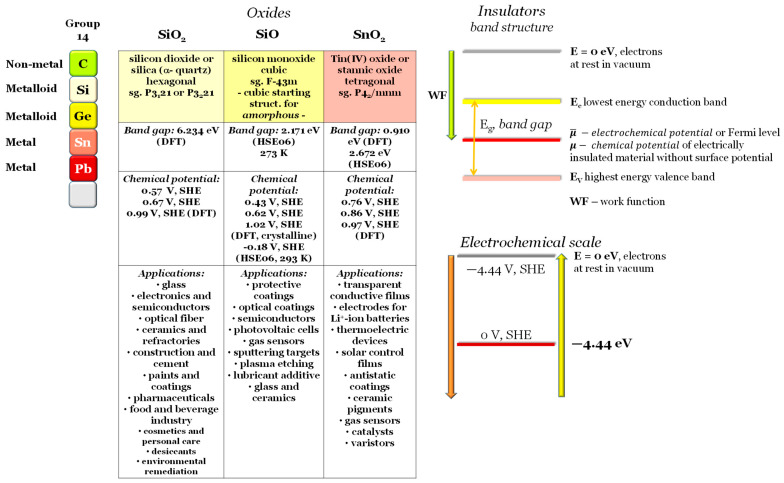
Electronic transport properties and applications of SiO_2_, SiO, and SnO_2_. Visual definition of *work function* (WF), lowest conduction band energy (E_c_), highest valence band energy (E_v_) and band gap (E_g_), electrochemical ($\bar{\mu }$) and chemical (μ) potentials. Electrochemical scale: standard hydrogen electrode (SHE) vs. physical scale where E = 0 eV is the energy of the electrons at rest on a surface in a vacuum. Note: all results are original to this work.

Silicon dioxide (SiO_2_), silicon monoxide (SiO), and tin dioxide (SnO_2_) are compounds of Silicon (Si) and Tin (Sn), elements of group 14 of the periodic table (Figure 1), each exhibiting distinct properties and applications. Silicon dioxide, commonly known as silica, is a fundamental component of the Earth’s crust and is utilized in various forms, from quartz crystals to glass manufacturing. Silicon monoxide is a less stable silicon oxide but finds applications in protective coatings, semiconductors, and nanotechnology. On the other hand, tin dioxide, or stannic oxide, is distinguished for its excellent conductivity and is employed extensively in the manufacturing of transparent conducting films, gas sensors, and optoelectronic devices (Figure 1). These compounds showcase the diverse range of properties and functions that oxide materials derived from Silicon and Tin can offer in scientific, industrial, and technological domains [1,2]. Hereafter, we present less studied electronic transport properties in conjunction with the chemical potentials (μ) that define how these oxides behave in heterojunctions; this latter property is crucial to understanding semiconductors or transistors, photovoltaics, and batteries, among other solid-state devices.

**Silicon dioxide, SiO_2_,** commonly known as silica, is one of the most abundant compounds on Earth. It comprises 99.5% SiO_2_, with a very small percentage of impurities in its natural state. It can be found in rocks, sand, quartz (various forms), opal, agate, and plants. Depending on how it was produced, silica comes in three different types: quartz or crystalline silica, diatomaceous silica (sedimentary rock), and vitreous (fused) silica. Silica is predominantly found as quartz, constituting 12–14% of the lithosphere, and is thermodynamically stable under natural environmental conditions.

Vitreous silica is fabricated by subjecting crystalline forms of silica to controlled high temperatures above 1996 K (1723 °C) and quickly cooled. Therefore, silica exists in crystalline (quartz) or amorphous forms such as vitreous silica [3,4,5,6].

Since the fifties, silicon dioxide has dominated microelectronics central to the global economy. However, lately, it has been substituted by HfO_2_, a ferroelectric oxide that allows a transistor, limited by Boltzmann’s tyranny to a minimum subthreshold swing $SS=k_{B}T\frac{ln\left(10\right)}{q}\approx 60\;mV/dec$, to become SS < 60 mV/dec. Far beyond the microelectronics field [7,8], it is present in optoelectronics (glass fibers, lasers) and acoustic electronics (quartz) [9].

Despite having a central role in electronic technology, it is also an essential source of raw materials, such as glass, construction materials, ceramics, food, catalysis, pharmaceuticals, and jewelry [3,9]. Silica is the base materials of the glass industry but is also used in concrete, being one of its main components. Thermal insulation is also frequently achieved using SiO_2_, allowing for a reduction in energy costs, contributing to the objective of reducing building energy waste and moving towards “zero energy buildings” [10,11].

The applications of SiO_2_ are vast: it is used in food as an additive, as an anti-caking agent (substances added to powdered or granulated foods to prevent them from forming lumps due to moisture absorption); and in the constitution of films for food preservation, combined with biodegradable polymers, improving their ductility qualities while maintaining the necessary thermal and mechanical properties. Some studies indicate it may also have anti-microbial and anti-oxidant effects [12,13].

The nanoscale variety of SiO_2_ has seen developments, with applications such as advanced catalysis, biomedical and environmental applications, wastewater treatment [14], nanocomposites, and thin-film transistors (TFTs) for applications in flat-panel displays [15,16].

Pure silica is white or colorless, although it can have color depending on impurities. It is odorless, tasteless, and shows insulating and dielectric properties and good thermal conductivity compared to other insulating materials.

**Silicon monoxide, SiO,** has gathered notoriety due to its unstable nature, amorphous structure (99.9%), oxygen content stoichiometry, and chemical disproportion compared to silicon dioxide (SiO_2_). Its application potential is such that new developments continue to be studied, patented, and launched into the market. In the focus of these developments lay the transistor or semiconductor industry, where the conduction mechanism observed in the insulator/semiconductor (SiO) includes the Poole–Frenkel emission originating donor levels in the forbidden band of SiO, instead of the Schottky emission from the electrodes (e.g., metals) in metal–insulator–semiconductor heterojunctions. The Poole–Frenkel emission process is primarily voltage-dependent, and its frequency dependency is generally weak. Additionally, the hopping mechanism aligns with the amorphous nature of silicon monoxide [17].

Silicon monoxide is a chemical compound where Silicon is present in an oxidation state +2. SiO in its solid state is yellowish-brown and is an electrical and thermal insulator. However, Briner and Landolt [18] show that SiO has a band gap of approximately 2 eV and is a semiconductor material. Sritharathikhun et al. [19] state that an optimal energy bandgap for the upper cell in a multijunction solar cell should be about 1.8–2.0 eV. Thus, the intrinsic amorphous silicon oxide film can have applicability as a superior cell absorber in triple-junction solar cells and the thin films of solar cells. They point out that the advantage of this intrinsic amorphous SiO film is its absorption capacity in the region of short wavelengths, due to its relatively wide bandgap. Microcrystalline SiO has also been shown to be more promising than microcrystalline SiC for application in photovoltaics, both as high-efficiency amorphous in the upper cell and as microcrystalline in the lower cell [20].

The relative dielectric constant of SiO is 5.3, which has been shown to be higher than that of SiO_2_ (3.9). The thermally evaporated SiO or SiO_x_ are excellent dielectrics for graphite-based devices [21].

Li et al. [22] studied the SiO in magnetic thin films because of their growing interest in magnetoresistance (MR) research and applications in ultra-high-density magnetic storage fields, magnetic sensors, and magnetic keys. The authors analyzed the growth of a Co film for application in high magnetic fields (HMF) in conjunction with SiO doping to adjust the surface particles’ size and morphology. They concluded that SiO could suppress the abnormal aggregation of the Co particle on the surface.

The use of SiO/C and SiO_x_/C as an anodic material in lithium-ion batteries (LIBs) has been investigated [23,24,25] because of its high theoretical capacity, small volume expansion, and longer life than graphite and Silicon, yielding batteries with higher capacity and good stability. Pan et al. [26] performed a systematic comparative study on the electrochemistry of Si and SiO anodes. Ge et al. [27] showed the possibility of manufacturing porous SiO with a carbon coating at low temperatures. The growth of vertical graphene in SiO microparticles was also achieved [20], resulting in a better electrical conductivity of SiO, notably at the single particle level and electrode level, but also providing Li-ion transport channels. Anode composite materials for lithium-ion batteries were prepared to include SiO, namely bm-SiO/Ni/rGO (where bm is ball milling and rGO is reduced graphene oxide) and SiO-Sn_x_Co_y_C_z_, respectively [28,29].

Generally, SiO has been applied as: (1) Protective Coatings for a wide range of surfaces showing good barrier properties against oxidation and moisture, making it a valuable choice for protecting metals, ceramics, among others, against corrosion [30]; (2) Optical enhancements—thin films of SiO find application as optical coatings enhancing reflectivity and reducing glare in optical components such as lenses, mirrors, and related elements [31]; (3) Semiconductor fabrication—SiO plays a pivotal role in photolithography processes within the semiconductor industry, primarily in mask-making and acts as a light-sensitive layer while producing microcircuits and other semiconductor devices [32]. (4) Photovoltaics—SiO contributes to the advancement of solar cell technologies by minimizing surface reflections and enhancing light absorption leading to increased solar cell efficiency [33]. (5) Nanotechnology applications—SiO nanoparticles hold promise in the field of nanotechnology as they can be utilized in the fabrication of nanostructured materials and devices, contributing to the development of cutting-edge technologies [34]. (6) Glass manufacturing, ceramics and composites, and lubrication enhancement—SiO is a critical component in the production of specialized glass; it imparts unique properties such as adjustments to its refractive index and hardness, making it ideal for specific applications like optoelectronics and fiber optics [35]. As a precursor material, SiO is vital in synthesizing advanced ceramics and composites, imparting specific qualities to the final products, such as improved mechanical strength and thermal stability [36]. SiO can function as an additive in lubricants, effectively reducing friction and wear in machinery and engines and enhancing their performance and longevity [37].

**Tin(IV) dioxide, SnO_2_**, possesses some unique characteristics that make it applicable in many fields, some of them familiar to SiO_2_ and SiO, as expected since they are oxides from the same group 14, and such as (1) optical components, (2) photovoltaics, (3) catalytic support materials, (4) solid-state chemical sensors, and (5) Li-ion batteries [38,39,40,41].

This variety of applications can be attributed to SnO_2_ being an n-type semiconductor with an energy bandgap between 3.4 eV and 3.7 eV [42], that while most commonly found in a tetragonal rutile structure P4_2_/mnm, cassiterite, may assume other structures at higher pressures such as CaCl_2_-type, α-PbO_2_-type, pyrite-type, ZrO_2_-type orthorhombic phase I, fluorite-type, and cotunnite-type orthorhombic phase II [41,43,44].

One of the key aspects to understanding SnO_2_ is its surface properties, which can be attributed to tin’s dual valency. This dual valency allows for a reversible surface composition transformation from stoichiometric surfaces with Sn^4+^ cations into a reduced surface with Sn^2+^ cations, depending on the chemical potential of the oxygen in the system. The reduction of the surface forms filled Sn 5s surface states in the band gap, modifying the electronic structure of the surface and decreasing the work function [45].

SnO_2_ has recently gathered attention as a next-generation anode material for Li batteries, replacing graphite-based anodes with a lower theoretical capacity, 372 mAh·g^−1^. The high capacity of Sn-based anodes can be attributed to two reactions: (1) the conversion reaction (${SnO}_{2}+4{Li}^{+}+4e^{-}⇄Sn+2Li_{2}O$, 711 mAh·g^−1^) and (2) the alloying/dealloying reaction ($Sn+4.4{Li}^{+}+4.4e^{-}⇄{Li}_{4.4}Sn$, 782 mAh·g^−1^) for a total of 1493 mAh·g^−1^. Nevertheless, the conversion reaction is generally irreversible, and the alloying/dealloying reaction is associated with a significant volume change [46].

## 2. Results and Discussion

**Silicon dioxide, SiO_2_.** Silicon and SiO_2_ were the semiconductor and photovoltaic technology base for many decades, remaining in numerous state-of-the-art applications; it is, therefore, useful to determine their electrical transport properties, although they have been the focus of many studies since the advent of transistors.

Although the Fermi level and energy of the band gap of the bulk hexagonal crystalline α-SiO_2_ have been extensively studied due to the abundance of Si and the many applications of SiO_2_, studies of its work function (WF) and charge carrier mobility are not as abundant.

**Figure 2:**
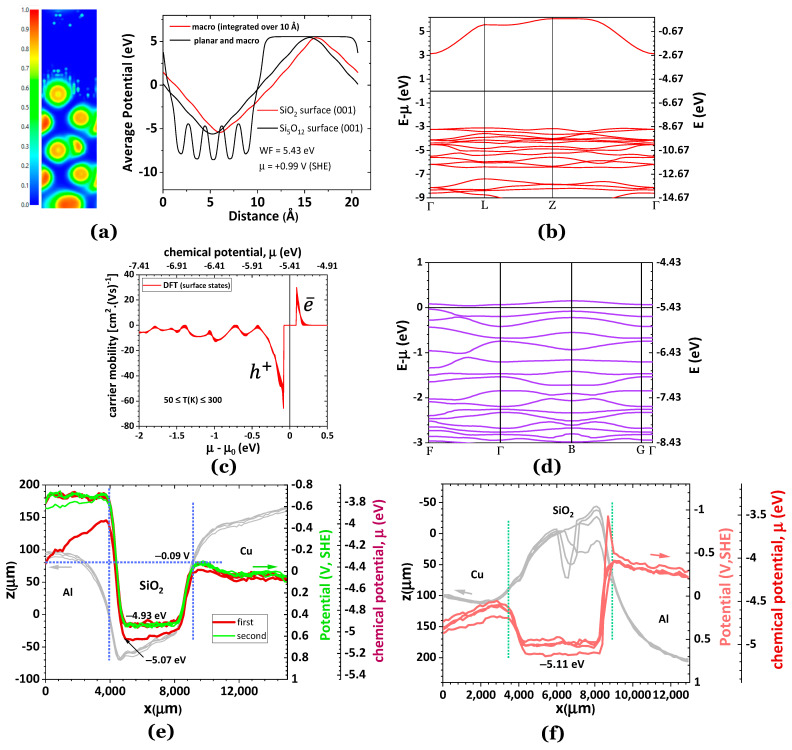
Potential and electrical properties of bulk and surface of SiO_2_ (alpha-quartz, hexagonal structure P3_1_21). (**a**) (001) The surface of SiO_2_ showcasing the electron localization function (ELF = 1 corresponding to dynamic localization and ELF = ½ corresponding to the electron gas) and the average potentials of SiO_2_ and Si_5_O_12_ demonstrating similar work functions and chemical potentials (for electrically insulated materials without self-surface potential contribution); (**b**) the electronic bulk band structure of SiO_2_ (showing insulating characteristics); (**c**) charge carrier mobility for 50 ≤ T(K) ≤ 300, showing insulator-like behavior, especially for the electrons, and the characteristics of semiconductor surface states (**d**); (**e**) experimental topography (grey lines) and SKP analysis of a Al/SiO_2_/Cu cell in which the Al is connected to the SKP circuit; the Cu contacts electrically with the cell, through the SiO_2_; (**f**) experimental topography (grey lines) and SKP analysis (red lines) of another Cu/SiO_2_/Al cell in which the Cu is connected to the SKP circuit; the Al contacts electrically with the cell through the SiO_2_; a slight tendency to perform as a polar dielectric with a center charged more negatively than the surfaces at the interfaces with Cu and Al, is observed at the SiO_2_ inner layer; a negative charge accumulation is noticed at the interface SiO_2_/Al, the only experiment for the SiO_2_ that reported such a sharp soliton in this system. Note: arrows point to the correspondent axis and vertical dotted lines mark the interfaces.

**Figure 3:**
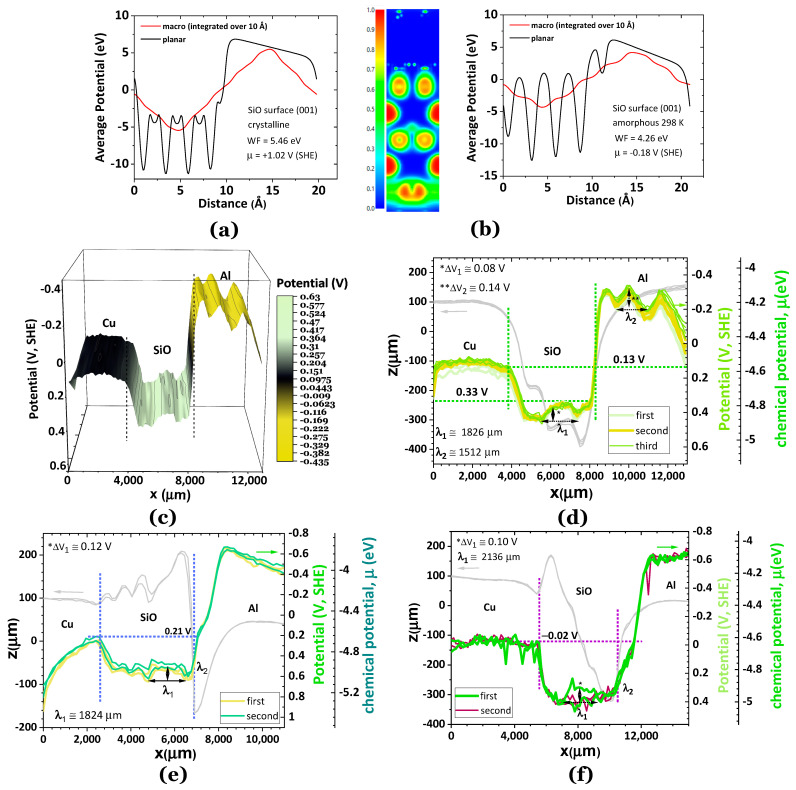
Electric potential properties of the bulk and surface of SiO (cubic crystalline, s.g. F-43m, and amorphous structure at 298 K). (**a**) Average potential for the (001) surface of SiO (crystalline) showing a WF of 5.46 eV; (**b**) electron localization function ELF (ELF = 1 corresponding to dynamic localization and ELF = ½ corresponding to the electron gas) and average potential for the (001) surface of SiO (amorphous at 298 K) showing a WF = 4.26 eV; (**c**,**d**) experimental topography (grey lines) and SKP analysis of a Cu/SiO/Al cell in which the Cu is connected to the SKP circuit; the Al contacts electrically the cell through SiO; a polar dielectric p-type semiconductor tendency is observed at the SiO layer, with a standing negative wave and a potential line slightly shifted up (accumulation of negative charges) at the Al side; the SiO incident wavelength is λ_1_ = 1826 μm; the Al transmitted wavelength, λ_2_ = 1512 μm; the amplitudes of the incident and transmitted waves are ΔV_1_ = 0.08 and ΔV_2_ = 0.14 V; (**e**) experimental topography (grey lines) and SKP analysis of another Cu/SiO/Al cell in which the Cu is connected to the SKP circuit; the Al contacts electrically with the cell through SiO; a polar dielectric n-type semiconductor tendency is observed at the SiO layer, with a standing negative wave and a potential slightly shifted up at the Cu side; the standing wave at SiO has a very similar wavelength than the cell in (**e**), λ_1_ = 1824 μm; at the SiO/Al interface a negative potential wave seems to be tunneling to the Al electrode; (**f**) an experiment of topography (grey lines) and SKP analysis performed one year later with the same SiO that was kept in the glovebox, shows approximately the same features as in (**e**) although with more positive SPP waves. Note: arrows point to the correspondent axis and vertical dotted lines mark the interfaces.

The theoretical WF was obtained with a novel method the group introduced [47,48]. The experimental WF was attained using a cell design that was also firstly optimized by the group [47,48] to determine the relative surface chemical potentials, the charge transport, SPP dynamics, and the tendency of the material to perform as an n- or p-types semiconductor, a polar dielectric, and a ferroelectric [49]. Figure 2a shows that the WF of SiO_2_ and Si_5_O_12_ are basically equal. Figure 2b–d show that SiO_2_ is an insulator with low electron mobility, even at the surface states (Figure 2c,d). The density of states (DOS) at the surface and the charge carrier mobility is higher for holes (h^+^) or cations. The latter is confirmed by the experimental SKP shown in Figure 2e; not only is the chemical potential of the inner SiO_2_ layer very similar to the theoretical in Figure 2a, showing an experimental error of ~6%, but there is also very slight mobility of the charge carriers that in Figure 3e seem to be cations moving in the direction of the Al, which is negatively charged at the surface with the SiO_2_ on the first surface scan. On the second scan of Figure 2e and in the experiment shown in Figure 2f, SiO_2_ shows a flat horizontal potential typical of an insulator, although with a small capability to accumulate negatively charged carriers at the inner layer of SiO_2_, by the movement of the positive charge carriers to the interfaces with Al and Cu. However, in Figure 2f, a negatively charged soliton is observed at the interface SiO_2_/Al that was not seen in any of the other experiments performed with SiO_2_, and that is likely a plasmonic soliton with origin on the Al. The experimental chemical potential of SiO_2_ in Figure 2f is about the same as in Figure 2e, showing an error of 6% in comparison with the theoretical.

**Silicon monoxide, SiO.** Silicon monoxide shows a marked dipolar behavior with an accumulation of negatively charged species away from the interface in the surface of the SiO (Figure 3) as in a standing wave with a characteristic wavelength that may be obtained from the SKP scans. Although, in Figure 3f, we are stretching our capacity to make a reliable determination of the wavelength due to the potential signal noise, the purpose makes sense within all the experiments performed, and the fact that the interval of measurements was as far as one year and corresponding to different cells and tip probes.

The crystalline and amorphous theoretical values for the WF (crystalline) = 5.46 and WF (amorphous) = 4.26 eV, respectively (Figure 3a,b), differing by 22%. Their average varies by 3% in the experimentally obtained data in Figure 3e,f but matches the WF obtained from Figure 3d.

**Figure 4:**
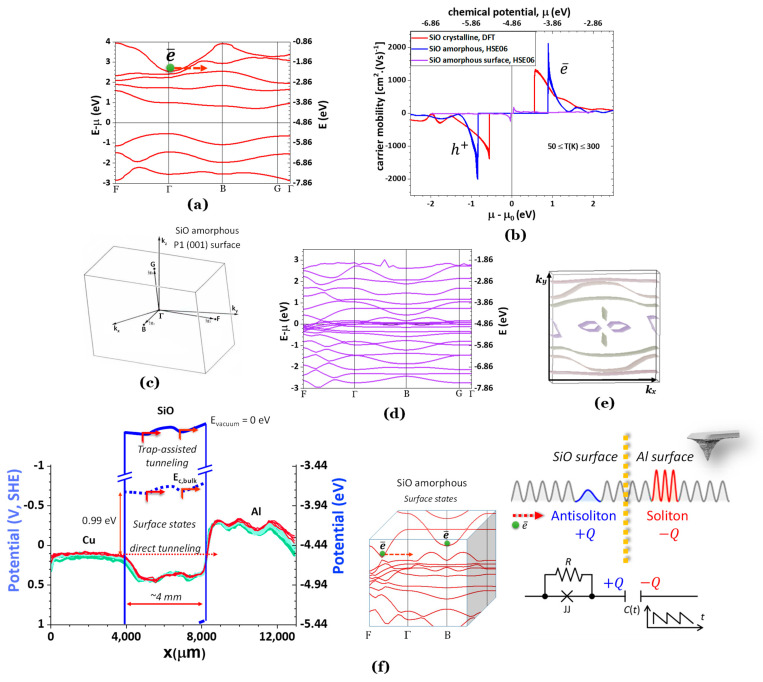
SiO (amorphous at 298 K) electrical properties. (**a**) Electronic bulk band structure showing hypothetical indirect trap assisted tunneling; (**b**) charge carrier mobility for 50 ≤ T(K) ≤ 300, showing a high electron mobility for μ(SiO) = −3.97 eV (amorphous, obtained using HSE06 hybrid functional) and −4.30 eV (cubic structure F-43m, DFT) which corresponds to the lower conduction band energy ~*E*_*c*,*min*_ and that same energy is obtained experimentally by SKP in Figure 3; the carrier mobilities correspond to those of a metal at ~*E*_*c*,*min*_; (**c**) the Brillouin zone for a calculated P1 triclinic structure as the symmetry of the SiO structure had to be lowered for performing the AMD simulations to obtain the amorphous structure at 298 K; (**d**) surface states for SiO amorphous with a corresponding Fermi surface at (**e**); (**f**) hypothetical schematics of the electronic band structure (blue lines) showing Poole–Frenkel emission/trap-assisted tunneling mechanism (red arrows) on the SiO surface in a Cu/SiO/Al heterojunction cell assisted by the surface states (it is possible that this latter mechanism is an electron-hole = exciton tunneling mechanism as the mobility of the holes is equally large to the electrons); partial surface states in the Brillouin zone showing trapped electrons; the propagation of SPP may lead to the nucleation of soliton and anti-soliton pairs that may arise from the interaction Cu/SiO (trap tunneling assisted)/Al where the SiO/Al forms a modulated capacitor *C*(t), and the trap assisted tunneling at the surface of the SiO is described by a Josephson junction (inductive element) in parallel with a resistor, *R* (this schematic was adapted from [50], developed for another context). Note: the dotted yellow line marks the interface SiO/Al.

Another critical feature is the definition of the transmitted waves in the surface of the Al from the SiO potential well, as in SPP waves propagating in a heterojunction dielectric/metal (such as in quantum dots). The transmitted wave in the Al surface of Figure 4d is written as $\psi_{t}(x)=T\psi_{i}(x)$ where $\;\psi_{i}(x)$ is the incident wave, and $T=\frac{k_{t}{{|\psi }_{t}\left(x\right)|}^{2}}{k_{i}{{|\psi }_{i}\left(x\right)|}^{2}}$, where $k_{t}$ and $k_{i}$ are the wave numbers of the transmitted and incident waves, respectively. The transmitted wave is also a function of the impedances and follows $T=\frac{2Z_{1}}{Z_{1}+Z_{2}}$ where $Z_{1}$ is the impedance of the incident medium (SiO), and $Z_{2}$ the impedance of the metal (Al). For SiO and Al (Figure 3f), $k_{i,SiO}=34.4\;$cm^−1^, $k_{t,Al}=41.6\;$cm^−1^, and $T_{SiO/Al}=4.2$ corresponds to transmission in a quantum square-like potential (heterojunction SiO/Al). The wavelength spectrum matches the microwave region near the infrared, consistent with 10^10^ Hz to 1 THz frequencies. From the SKP spectra, f(SiO) ≈ 0.16 THz (considering the surface plasmon polariton SPP propagation on the surface of SiO having air as the dielectric). A relationship between the impedances of the Al and SiO is established, $T_{SiO/Al}=\frac{2Z_{SiO}}{Z_{SiO}+Z_{Al}}\Rightarrow Z_{Al}=-0.52\;Z_{SiO}$. This result shows a negative impedance for one of the materials, likely the Al [47,48].

Notably, the conditions for having a transmitted wavefunction higher than the incident wavefunction depend on various factors such as the shape and height of the potential barrier/well, the energy of the incident wave, and the quantum properties of the system. In Figure 3e,f calculations for the transmission may not be performed because Aluminum’s transmitted wave is not defined. However, the wavelength of SiO in Figure 3e, λ_1_ = 1824 μm is approximately the same as λ_1_ = 1826 μm in Figure 3d; the SPP frequency is also f(SiO) ≈ 0.16 THz.

Figure 4 shows the theoretical data for the electronic band structure (Figure 4a), carrier mobility $\mu^{*}$ (Figure 4b), and hypothetical mechanism for surface conduction in SiO, seconded by its surface states and Fermi surface (Figure 4c–e).

The Poole–Frenkel emission or, likely, trap-assisted tunneling happens at the surface of the SiO, allowing for negative resistance, overall resulting in electron transport from the interface with the Cu to the Al (Figure 4f). For this phenomenon to occur, polaritons that are quasiparticles ensuing from the strong coupling of photons with a dipole-carrying excitation are formed at the surface of the SiO. This latter surface transport is hypothesized to be described by the model in Figure 4f, in which a Josephson junction JJ in parallel with a resistor represents the trap-assisted electron transport on the surface of the SiO. At the interface with the Al, a double-layer capacitor is formed where the positive charge +Q corresponds to a nucleated anti-soliton on the surface of the SiO, and –Q the nucleated solitons on the surface of the Al, caused by the tunneling of electrons from the lowest chemical potential (Cu) to the highest (Al) against the drift direction assisted by the surface of the SiO. The resulting capacitance $C\left(t\right),$ or potential V(t), is modulated (Figure 4f). This process may require a negative refraction medium, herein not engineered.

We have only observed similar phenomena with a metal1/ferroelectric/metal2 cell. The model proposed in Figure 4f was adapted from [50], where surface acoustic waves induced a negative resistance state in superconducting NbSe_2_. Here, the waves are generated by the AC component of the potential in the SKP signal, responsible for the probe’s vibration.

In the scenario of Figure 4f, the dipoles are likely electron-hole pairs or excitons, and the cell that allows for this behavior is a cavity with two metallic quasi-mirrors (Cu and Al) separated by a quantum well (the surface of the SiO). The symmetrical and metallic-like mobility of both electrons and holes in Figure 4b, the electronic structure in Figure 4a, surface states, and the Fermi surface in Figure 4c–e seem to corroborate a Poole–Frankel/trap-assisted tunneling-like SiO surface conduction. Notably, the Poole–Frenkel emission was demonstrated before in a graphene/MoS_2_/SiO_x_/Ni [47,48] heterojunction beyond the amorphous surface of the SiO_2_ [47,48].

**Figure 5:**
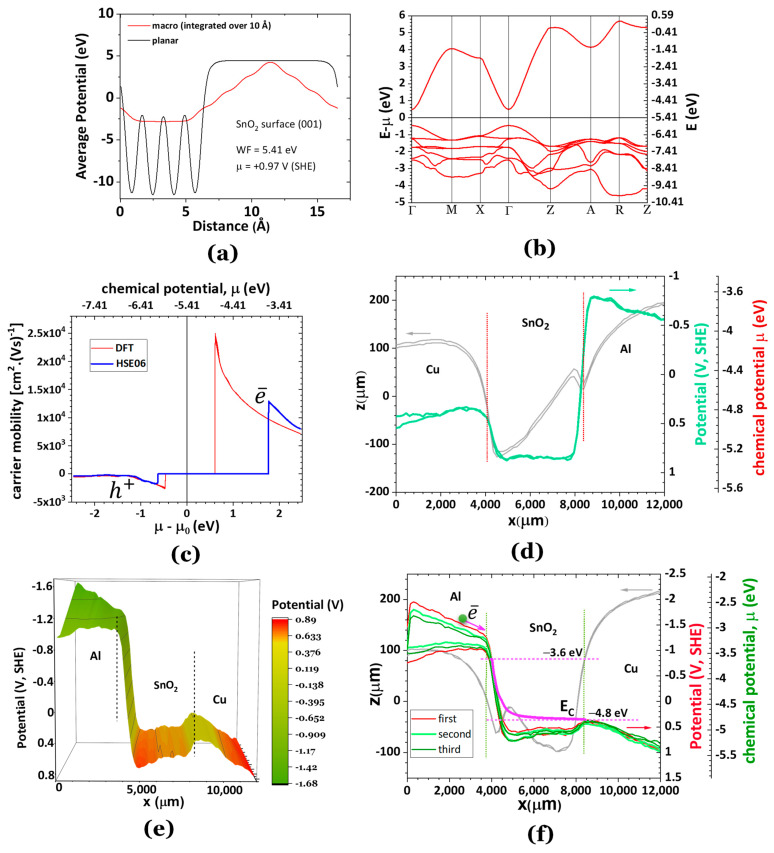
Potential and electrical properties of the bulk and surface of SnO_2_ (tetragonal structure P4_2_mnm). (**a**) Average potential for the (001) surface of SnO_2_ showing a WF of 5.41 eV; (**b**) electronic bulk band structure showing semiconductor characteristics; (**c**) charge carrier mobility showing extremely high electron mobility for μ(SnO_2_) = −3.64 eV (obtained using HSE06 hybrid functional) and −4.80 eV (obtained using DFT) which correspond to the lower conduction band energy ~*E*_*c*,*min*_; The carrier mobilities correspond to those of metal at ~*E*_*c*,*min*_; (**d**) experimental topography (grey lines) and SKP surface chemical potential (green lines) for Cu/SnO_2_/Al showing a flat chemical potential (−5.28 eV) in agreement with the theoretical WF (5.41 eV) and an insulator behavior; (**e**,**f**) experimental topography (grey lines) and SKP of an Al/SnO_2_/Cu cell showing typical n-type semiconductor behavior similar to ZnO [47,48] with an accumulation of negative charge at the SnO_2_/Cu interface giving rise to a reduced energy barrier or internal resistance, while on the Al/SnO_2_ interface the quantum barrier increases, although the Al surface potential helps the conduction of electrons towards the interface with SnO_2_. Note: E_C_ minimum energy of the conduction band of SnO_2_. The dotted lines denote the interfaces.

**Tin(IV) dioxide, SnO_2_.** Tin dioxide has been receiving recent attention as an anode for Li-ion batteries. It has also been applied as a wide-band semiconductor. Herein the chemical potential of electrically insulated SnO_2_ oxide was determined via the simulation of the average potential of a (001) SnO_2_ surface, and the calculation of the work function has shown that WF = 5.41 eV (Figure 5a). The chemical potential is therefore μ(SnO_2_)/e = +0.97 V, SHE ⇔ μ(SnO_2_) = −5.41 eV. The SKP experimentally obtained chemical potential is μ(SnO_2_) ≈ −5.3 eV (~±2%). The band structure, simulated using DFT, shows a band gap energy of 0.9 eV compared to 2.7 eV simulated using hybrid HSE06 (table in Figure 1). It is well known that DFT is often not the best method to determine the band gap energy of a semiconductor (Figure 5b), which also reflects on the carrier mobility vs. chemical potential, as the electrons have a step increase in their mobility at the minimum energy of the conduction band, *E*_*c*,*min*_ (Figure 5c). The carrier mobility of electrons (positive) reaches metal-like values.

While Figure 5d shows a perfectly flat SnO_2_ chemical potential, with SnO_2_ performing as a quantum well, with no plasmonic or SPP noticeable phenomena at the surface of Cu and Al metals and the SnO_2_ Figure 5e–f, however, shows an apparent bending of the surface chemical potential of the SnO_2_ to meet the chemical potential of the Cu with negative charge accumulation at the interface. This behavior of SnO_2_ configures an n-type semiconductor. The pink solid line in Figure 5f is proposed for the conduction band *E_c_*_,*min*_ following the electronic band structure (Figure 5b) and carrier mobility in Figure 5c.

**Figure 6:**
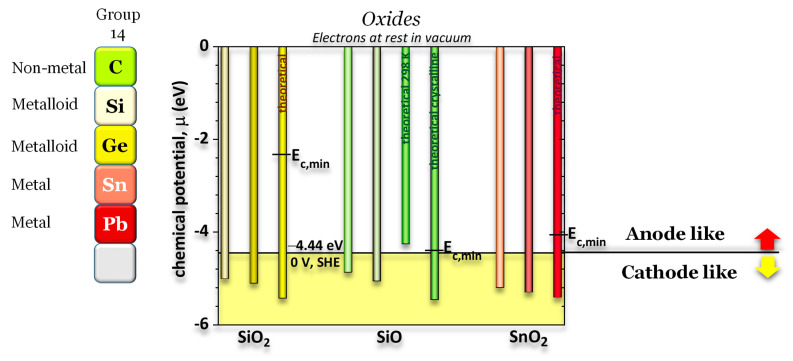
Comparison between the chemical potentials of the electrically insulated SiO_2_, SiO, and SnO_2_ showing similar values for corresponding crystalline structures. All theoretical chemical potentials or *work functions* for the oxides, except the SiO amorphous at 298 K, are similar and cathode-like, likely due to the groups’ common properties, bearding in mind that the work function measures the energy necessary to bring one electron from the Fermi level to the surface in vacuum (E = 0 eV). It shows that the internal electrical levels do not interfere considerably with the work function. The experimental chemical potentials are in good agreement with the simulated correspondents. For SiO, the experimental data matches the average between the amorphous at 298 K and the crystalline theoretical. The minimum energy of the conduction bands and the band gaps for SiO_2_, SiO, and SnO_2_ are different, with SiO_2_ being an insulator and SiO and SnO_2_ semiconductors.

**SiO_2_, SiO, and SnO_2_.** The three oxides demonstrate very similar chemical potentials, meaning that the internal electron levels do not contribute significantly to the oxide formation and *work function* as expected from its definition. However, their bands show very different structures with different energies for the lowest allowed state in the conduction band, indicating different electron affinities (*E_EA_ = E_vacuum_ − E_c_*_,*min*_*)*, especially between SiO and SiO_2_, as shown in Figure 6. All the oxides perform as cathode-like materials with a chemical potential μ < −4.44 eV.

Silica shows an experimental band gap of 8.9 eV and a *work function* of 5.4 eV [51], 5.0 eV—experimental [52], 5.2 eV—theoretical [53], which is in agreement with the simulated WF(SiO_2_) = 5.43 eV and the obtained by SKP WF(SiO_2_) = 5.1 eV. The band gap, as expected, was underestimated by the simulations E_g_ (SiO_2_) = 6.2 eV (table in Figure 1).

Silicon monoxide shows a band gap of approximately 2 eV [18] in agreement with the 2.17 eV obtained recurring to the hybrid functional HSE06 (table in Figure 1).

For SnO_2_, the simulated band gap is 3.67 eV [38] and the experimental band gap is 3.60 eV [54] for a work function of 5.92 eV [55]. Yet again, the simulations underestimate the band gap of 2.67 eV (HSE06) (table in Figure 1) but are in reasonable agreement with the work function 5.41 eV.

## 3. Materials and Methods

**Materials.** The oxides in this study used as dielectrics were powders of (1) Silicone oxide (IV) 99.5%, powder diameter < 44 µm from Thermo Fisher; (2) Silicon (II) oxide, optical grade, 99.8%, powder diameter < 10 µm (−325 Mesh) from Thermo Fisher; (3) Tin (IV) oxide 99.9%, powder diameter < 10 µm from Thermo Fisher. All oxides were kept and manipulated to fill the cell’s gap in a glovebox with an argon atmosphere with O_2_ and H_2_O < 1 ppm.

The cells were prepared using Al and Cu metal electrodes. These electrodes were positioned with a specific parallelepipedic gap filled with each oxide. The dimensions of the materials used in the Al/group 14 oxide/Cu cell were as follows: Aluminum (11 × 20 × 4.7) mm^3^, oxide (5.5 × 22 × 4.7) mm^3^, and copper measuring (11 × 20 × 4.7) mm^3^ (Figure 2).

As stated, the oxide powders were introduced into the gap between the contact with the metals; the cell was supported by epoxy resin. The powders were hand-tooled and pressed against the cell, which was cleaned and polished after each experiment.

**Methods.** Allowing for the specific case where a species *i* are electrons, ${{\bar{\mu }}_{e}}^{\alpha }$ is the electrochemical potential or *Fermi level* and corresponds to the electron energy level. The electrochemical potential of species *i*, ${{\bar{\mu }}_{i}}^{\alpha }$, in a phase *α* with charge $z_{i}$, can be expressed,

(1) $${{\bar{\mu }}_{i}}^{\alpha }={\mu_{i}}^{\alpha }+z_{i}F{\phi_{i}}^{\alpha }$$

where ${\mu_{i}}^{\alpha }$ represents the chemical potential of *i* in the *α* phase, $F=eN_{A}$ is the Faraday constant equal to the charge of the electron times the Avogadro number, and ${\phi_{i}}^{\alpha }$ the surface potential of the *i* species, also in the *α* phase [47].

If two species, *i* and *j*, establish electrical contact (they do not have to be in physical contact), their electrochemical potential, ${\bar{\mu }}_{i}$ and ${\bar{\mu }}_{j}$, will reach an equilibrium ${\bar{\mu }}_{i}-{\bar{\mu }}_{j}=0$ where the electrochemical potential of both species will be the same, as represented by,

(2) $${\bar{\mu }}_{i}-{\bar{\mu }}_{j}=0=\left(\mu_{i}-\mu_{j}\right)-z_{i}F{(\phi }_{j}-\phi_{i})⟺\mu_{i}-\mu_{j}=z_{i}F{(\phi }_{j}-\phi_{i})$$

The Fermi level ${\bar{\mu }}_{i}$ corresponds to the chemical potential ${{\bar{\mu }}_{i}}^{\alpha }={\mu_{i}}^{\alpha }$ only if the material is electrically isolated and does not produce self-surface potential; in other words, if ${\phi_{i}}^{\alpha }\approx 0$.

**Figure 7:**
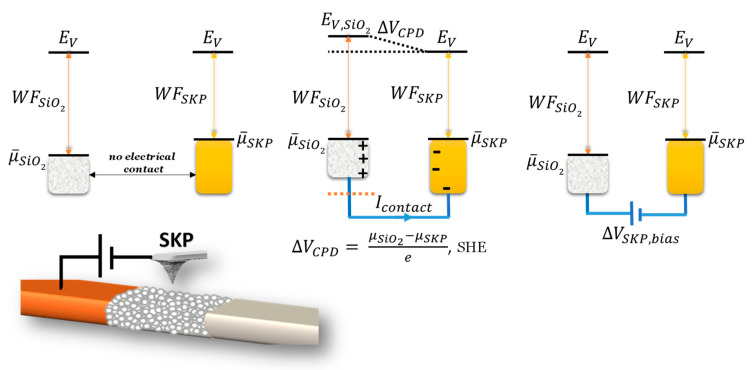
Example of a working Cu/SiO_2_/Al cell and corresponding SKP working principles when SiO_2_ is being scanned. The alternating current (AC) working components responsible for the vibration of the probe is not featured in the schematics. Note: *E_V_* = vacuum energy, *WF* = work function; Δ*V_CPD_* = contact potential difference.

Equation (2) reveals what will happen at the metal–oxide interfaces. This information will be tested and analyzed using a cell like the one shown in Figure 7 and a scanning Kelvin probe (SKP). The horizontal cell allows for determining the work functions or surface chemical potentials at the bulk materials and interfaces of the materials constituting the cell. It also allows us to understand how the electrical and ionic transport is made throughout the cell to equilibrate the Fermi levels spontaneously, how surface plasmons polaritons (SPP) waves travel through the interfaces air–metal, air–oxide, and oxide–metal (indirectly), the quantum barriers or wells within the cell, and to determine the chemical potential in the bulk material within a thick layer d > 4 mm.

The SKP is a non-invasive technique derived from atomic force microscopy (AFM). It allows the electrochemical study of the materials’ surface, especially conductors and substances that are electrically active.

The SKP was used to determine the *work function* of the electrodes, the oxides, and the latter materials while performing in the cell. The equalization of the sample and probe Fermi levels [56] (Figure 7) takes place after the formation of a capacitor between the surfaces, which is subsequently eliminated by the application of a bias voltage ΔV_CPD_ equal to ΔV_SKP_. It is noteworthy that the probe of the SKP oscillates with a specific frequency, ω, and consequently, the bias applied voltage is the result of a DC with an AC component.

The working principle of the SKP is our focus, and we bring a novel approach to the study of energy harvesting, storage, and switching devices [56,57,58].

The SKP analyzes, therefore, the surface’s contact potential difference (CPD), leading to the calculation of the work functions (WF). The capacitive tracking measurement (CTM) was used to scan the surface topography. A Biologic SKP-M470 with U-SKP370 probe tips of W-wire with diameters of 500 µm and 150 µm was used to perform SKP analysis [59]. The CTM/SKP and the cell circuit are closed through the positive (Cu) or negative (Al) electrodes, but never both simultaneously.

In summary, with the SKP analysis, it was possible to attain: (1) the materials’ topographies; (2) contact potential differences within a cell; (3) the *work function* of each material in the cell; (4) the bulk chemical potentials away from the interfaces; and (4) *in operando* behavior of a solid-state cell. The SKP measurements were performed at 25–35 °C in a dry box with a dew point ≤ 0 °C.

**Simulations.** Density functional theory (DFT), as implemented in VASP [57] and MedeA [59], was used to simulate the crystal structures of SiO_2_, SiO, and SnO_2_. The conditions used for the simulations were DFT with exchange-correlation GGA-PBE. For SiO_2_, SiO, and SnO_2_, additional hybrid functional HS06 was also used, as these materials showed semiconductor or insulator characteristics. The simulation parameters were cutoff planewaves > 400 eV, projection in the reciprocal space, and 0.1 or 0.2 Å spacing k-points. The three oxides were designed as surfaces, with a minimum gap of 10 Å to avoid the periodic interference of the lattice. The surface allowed the calculation of the total local potential. The average potential was integrated over 10 Å. The work function was calculated from the total local potentials by finding the Fermi level as the medium value between the minimum and maximum macro-averaged potential. This value should be a minor correction to the zero-point Fermi level, but this correction is relevant for some materials. The *work function* is the energy difference between the maximum (electron at rest in vacuum) and the Fermi level (Figure 1) [57].

Ab initio molecular dynamics (AMD) was employed to simulate a closed system, which was thermostatted within a heat bath at a constant temperature and pressure, referred to as NP’T conditions. The system of interest was the optimized crystalline structure of SiO, initially cubic F-43m space group. Before the AMD NP’T simulation at 298 K, a microcanonical simulation NV’E was conducted at 2000 K; the system was then quenched to 298 K (25 °C).

While simulating the crystalline precursor structures, energy of formation, internal pressure, and trajectories were calculated and controlled to guarantee that the computed structure was stable. After the optimization of atomic sites, volume, and shape, the Boltzmann transport properties [60], Fermi surface, and electronic transport properties were simulated for both the bulk (crystalline and amorphous) and the surface: band structure, electron localization function, and charge carrier mobility (herein positive for electrons and negative for holes), as a function of the chemical potential and temperature.

The Boltzmann transport theory [49,61,62] was employed, and the energy-dependent electrical conductivity tensor was expressed as a function of the energy E as [60],

(3) $$\sigma_{\alpha \beta }\left(E\right)=\frac{e^{2}}{\Omega_{C}}\sum_{k}\sum_{n}-\frac{\partial f(E)}{\partial E}v_{kn}^{\alpha }v_{kn}^{\beta }\tau_{kn}$$

where $f(E)=\frac{1}{e^{\beta (E-\mu )}+1}$ where $\beta =1/k_{B}T$ and μ the chemical potential, $\Omega_{C}$ the volume of the cell, $\tau_{kn}$ is the relaxation time that reflects the electron-phonon scattering on the electronic states and is dependent on the band index (n), spin, and k-point, $v_{kn}^{\alpha }$ is the Cartesian component of the group velocity and is given by,

(4) $$v_{kn}^{\alpha }=\frac{1}{\hbar }\frac{\partial \epsilon_{kn}}{\partial k_{\alpha }}$$

where the inverse of the mass tensor is given by:

(5) $${\left(\frac{1}{m_{n}^{*}}\right)}_{\alpha \beta }=\frac{1}{\hbar^{2}}\frac{\partial^{2}\epsilon_{kn}}{\partial k_{\alpha }\partial k_{\beta }}$$

the curvature of a band at a particular k-point. The charge carrier mobility is directly proportional to the inverse transport mass (5).

Carrier mobility $\mu^{*}$ is related to Equation (3) as $\sigma =n^{*}e\mu^{*}$ where $n^{*}$, the charge carrier density, represents the number of charge carriers (usually electrons) per unit volume. It is a property of materials that describes the ability of charge carriers (such as electrons, holes, or cations) to move through the material in response to an electric field. The mobility of charge carriers $\mu^{*}$ may vary significantly depending on the material and the specific conditions. **Metals:** In metals, the carrier mobility is relatively high, 1000 ≤ $\mu^{*}[cm².{\left(Vs\right)}^{-1}$] ≤ 100,000. **Semiconductors:** The mobility of charge carriers in semiconductors is intermediate between metals and insulators. Intrinsic semiconductors typically have mobilities of 100 ≤ $\mu^{*}[cm².{\left(Vs\right)}^{-1}$] ≤ 1000. **Organic Semiconductors:** Organic semiconductors used in organic electronics can have carrier mobilities, 0.01 ≤ $\mu^{*}[cm².{\left(Vs\right)}^{-1}$] ≤ 10. These materials have lower mobilities compared to inorganic semiconductors. **Amorphous Semiconductors:** Amorphous or disordered semiconductors often have lower mobilities than their crystalline counterparts. Mobility values, 0.001 ≤ $\mu^{*}[cm².{\left(Vs\right)}^{-1}$] ≤ 1. **Two-Dimensional Materials:** Two-dimensional materials like graphene and transition metal dichalcogenides (e.g., MoS_2_, WSe_2_) can exhibit exceptionally high carrier mobilities. Graphene, for example, can have mobilities exceeding $\mu^{*}[cm².{\left(Vs\right)}^{-1}$] ≥ 10,000. **Insulators:** Insulators have low carrier mobility, typically $\mu^{*}[cm².{\left(Vs\right)}^{-1}$] < 10. In insulators, charge carriers have low mobility due to the large energy gap. It is important to note that these values are approximate and can vary depending on temperature, impurities, defects, and specific measurement conditions.

Herein, we have calculated the charge carriers mobilities of the semiconductors SiO_2_ (surface states), SiO, and SnO_2_ to understand the transport in the Cu/SiO_2_/Al, Cu/SiO/Al, and Al/SnO_2_/Cu cells.

## 4. Conclusions

The chemical potentials and electronic properties, such as band structure, carrier mobility, Fermi surface, and electron localization functions, were simulated for three oxides of group 14: SiO_2_, SiO, and SnO_2_. The chemical potentials were also determined for the bulk and surfaces of the materials while performing in operando in a Cu/oxide/Al cell. The experimental results are in excellent agreement with the theoretical and show a robust method to understand the redox character of a material when performing in a heterojunction. Therefore, the method may be used to match different kinds of materials for tailor-like performance in solid-state devices. The method also gives coherent information on the nature of the interface impedances, with well- and barrier-like roles for the insulator/semiconductor interlayers. In this study, the formation of solitons was also shown, and it results from trap-mediated surface transport through the oxides and strong coupling of photons with a dipole-carrying excitation leading to electron tunneling to the metal at the highest chemical potential (negative resistance).

The semiconductor type may also be perceived by surface chemical potential bending towards the adjacent metal in a multijunction, reflected on the SKP surface scan. The dipolar character with the formation of charged inner ‘domes’ is also distinctive in certain insulators and corresponds to higher dielectric constants. Here, we have determined the n-type behavior of SnO_2_ and the dipole-like character of SiO with a marked tendency to form negatively charged ‘domes’ that, in the model proposed, are anti-solitons.

SPPs have found applications in various fields, including surface-enhanced spectroscopy, plasmonic waveguides, optical sensing, and imaging. New ways to harness the unique properties of SPPs are being pursued for advancing technologies in optics, photonics, and nanoscale science; they may now count on SiO as an agent to implement those properties.

The Al surface chemical potential’s variable and even modulated character, assuming a wide range of possible values, was again observed even when the metal is not physically connected to the SKP but interacts through the semiconductor.

It was also possible to understand the dynamics of the spontaneous efforts to reduce the energy to a minimum in the multijunction (e.g., Al/SiO_2_/Cu) while equalizing the Fermi levels by dipolar character, then a p-type behavior, and finally a quantum well, with no visible charge transport.

## Data Availability

Data available on reasonable request.
